# Influence of psoriasis on circulatory system function assessed in echocardiography

**DOI:** 10.1007/s00403-015-1586-7

**Published:** 2015-06-28

**Authors:** Sylwia Milaniuk, Aldona Pietrzak, Barbara Mosiewicz, Jerzy Mosiewicz, Kristian Reich

**Affiliations:** Department of Internal Medicine, Medical University, Staszica St 16, Lublin, Poland; Department of Dermatology, Venereology and Paediatric Dermatology, Medical University, Radziwillowska St 13, Lublin, Poland; Students Medical Association, Medical University, Staszica St 16, Lublin, Poland; Dermatologikum Hamburg, Stephansplatz 5, Hamburg, Germany

**Keywords:** Psoriasis vulgaris, Psoriatic arthritis, Diastolic disorders, Aorta stiffness

## Abstract

Psoriasis vulgaris is a chronic disease with a multifactorial pathogenesis. It affects about 2–4 % of the population all over the world. In course of psoriatic arthritis, joints’ damages are observed. In patients with psoriasis vulgaris and psoriatic arthritis, there is increased morbidity and mortality caused by cardiovascular diseases observed. The aim of the study is to analyze the echocardiography of patients with psoriasis vulgaris and psoriatic arthritis on the basis of the literature available in PubMed database. Abnormalities found in echocardiography of patients with psoriasis include valvular defects (40.7 % of the patients), left ventricle diastolic dysfunction (27.8 %), and left ventricle hypertrophy (11.1 %). Left ventricle’s systolic disorders, increased aorta stiffness index and increased pulmonary artery blood pressure were also observed in this group of patients.

## Introduction

Psoriasis is a chronic disease with a multifactorial pathogenesis. Its symptoms may be noticeable on the skin, they also affect joints, and the inflammatory process manifestations are observed outside the skin [3, 24, 27]. Dermatologists as well as cardiologists have been studying cardiologic complications of psoriasis for many years [3, 24, 27, 44]. The reason for an increased cardiovascular morbidity in psoriasis seems to be the chronic systemic inflammatory process [7, 34, 35]. What is more, a number of studies and observations confirm an increased occurrence of the standard atherosclerosis risk factors and/or metabolic syndrome as well as cardiovascular diseases in patients with psoriasis [9, 18, 22, 27, 35, 45]. Current state of knowledge does not allow to determine if commonly known ischemic heart disease risk factors are also psoriasis risk factors or if they occur in course of disease and if psoriasis alone is heart diseases risk factor. Last hypothesis is supported by the fact that there is an increased intima-media complex thickness of the carotid artery and decreased nitroglycerin-induced dilatation of the brachial artery observed in patients with psoriasis after excluding diabetes, hypertension, renal insufficiency and cardiovascular diseases in their medical history. A significantly decreased flow-mediated vasodilatation, FMD, was observed in patients aged 23 ± 6 years with mild and moderate psoriasis after excluding coexistence of diseases affecting the vascular system [42]. The psoriatic march contains: immunological dysregulation, inflammation, insulin resistance, endothelial dysfunction, atherosclerosis, cardiovascular disease [7, 16, 35].

The aim of the study is to analyze the echocardiography of patients with psoriasis vulgaris and psoriatic arthritis referring to the available literature. The study is based on the analyzed publications from PubMed database using the search terms “psoriasis” (Mesh) and “echocardiography” (Mesh). We found 27 articles in English language dated between 1991 and 2014 and 3 articles from 1979 to 1986. We identified 17 original studies, which we selected for this review. First works concerning the cardiovascular system disorders in patients with psoriasis were published in the late 1980s. Table 1 shows the most important results of the analyzed studies.

**Table 1:** Echocardiography results in psoriatic patients (literature overview)

	LVDD, LVSD	LAD	EF	IVRT, DT, E/A	SPAP	Valvular defects	ASI	Duration of psoriasis mean/range (months)	PASI range/mean	Age mean (years)
Saricaoglu et al. [37]	↑	nd	↓	↑	nd	nd	nd	12–360	nd	48
Przepiera-Będzak et al. [33]	nd	nd	nd	↑	nd	+	nd	87	nd	50.7
Gonzalez-Juanatey et al. [15]	−	↑	−	−	↑	+	nd	84	nd	nd
Ardic et al. [2]	−	nd	−	−	nd	nd	↑	117.6	13.0	36.3
Gullu et al. [16]	−	nd	−	↑	nd	nd	nd	130	22.2	36.4
Karadag et al. [21]	−	nd	−	−	nd	nd	nd	nd	1.8–34	40.4
El-Mongy et al. [12]	−	−	−	−	−	−	−	151.2	29.1	51
Shang et al. [39] (2 groups of patients)	−	nd	−	↑	nd	nd	nd	nd	nd	44.5/50.6
Biyik et al. [6]	↑	nd	nd	↑	nd	+	nd	104	1.8–60	35
Gunes et al. [17]	↑	nd	nd	↑	↑	nd	nd	123.2	0.4–34	nd
Bicer et al. [5]	nd	nd	−	nd	nd	nd	↑	213	12.9	37
Zhao et al. [43]	−	nd	−	−	nd	nd	nd	188.4	15	46
Simsek et al. [40]	nd	nd	nd	↑	nd	nd	nd	129.4	0–34	35.8
Rowe et al. [36]	nd	nd	nd	↑	nd	nd	nd	132	nd	41
Pines et al. [31]	nd	nd	nd	nd	nd	+	nd	129.6	nd	46.5
Shang et al. [38]	nd	nd	nd	nd	nd	nd	↑	128.4	1–8	34
Osto et al. [28]	−	nd	−	−	nd	nd	nd	nd	nd	37

(↑), increase of parameter; (↓), decrease of parameter; (+), present valvular defects; (−), no changes between groups; LVDD (mm), left ventricular end-diastolic diameter; LVSD (mm), left ventricular end-systolic diameter; LAD (mm), left atrial diameter; nd, no data; IVRT (ms), isovolumic relaxation time; DT (ms), deceleration time; E/A ratio, early (E) to late (A) ventricular filling velocities ratio; SPAP (mmHg), systolic pulmonary artery pressure; ASI, aortic stiffness index; EF (%), ejection fraction

## Epidemiologic data and pathogenesis

Psoriasis is a chronic skin disease. It is one of the most common dermatoses and it is estimated that it occurs in about 2–4 % of the entire population [23]. Many studies and clinical observations show the increased standard cardiovascular diseases’ risk factors occurrence such as smoking, hypertension, glucose tolerance disorders and diabetes, overweight, obesity in this group of patients [9, 18, 22, 23, 27]. An issue of psoriasis alone as a cardiovascular disease risk factor is being more frequently discussed nowadays. However, there are no unequivocal data on this subject. Results of the studies show that there is an overexpression of various pro-inflammatory cytokines, such as interleukins (IL-1, IL-6, IL-8, IL-12, IL-15, IL-17-20, IL-22, IL-23), TNF-α and interferon-γ in psoriasis in the skin as well as in the entire organism [30]. What is more, cytokines’ secretion is observed to be constant [19]. These cytokines affect cellular components of the inflammatory infiltration within psoriatic plaque as well as the hyperproliferation of keratinocytes. In the blood serum in psoriasis vulgaris and psoriatic arthritis, there was a higher level of inflammation markers, such as CRP, fibrinogen, haptoglobin, complement fractions: C3, C4 observed [46]. There are also adipokines’ system disorders in blood, skin and adipose tissue [14, 39], disorders in the antioxidative system (MDA,ox-Ldl, TBA, anti-OxLDL antibodies, dismutase, glutathione peroxide, catalase) observed [34]. It is known that TNF-α as well as its receptors have a toxic influence on the cardiomyocytes [41]. What is more, an increased concentration of interleukin 17 occurred in unstable coronary disease and unstable angina pectoris. Recently, scientists have found the resemblance between psoriasis plaques and atherosclerosis plaques structure. It turned out that in both of them, there are activated Th1 and Th17 cells in similar proportion. Th1 line cells are characterized by an increased secretion of inflammatory cytokines, such as TNF-α, INF-γ, and IL-6, which cause the endothelial cells dysfunction. Th17 cells produce IL-17, which plays an important role in psoriasis pathogenesis and initiation of the inflammatory process within various tissues and organs [26]. An increased level of this interleukin was also observed in the serum of patients with unstable angina pectoris and in course of acute heart infarction [20]. Circulating IL-18 is prospectively and independently associated with CVD risk.

In about 5–20 % of patients with psoriasis, there is damage of joints observed [37]. In this group of patients, like in patients with psoriasis vulgaris, there is higher occurrence of conventional cardiovascular risk factors observed [9, 18, 22, 27]. Cardiovascular diseases are the main cause of mortality among these patients [25, 32]. It is estimated that risk of death in patients with psoriatic arthritis is 1.3 times higher compared to the general population [33].

One of the most important diagnostic methods in heart and vessels diseases is echocardiography, which enables to examine morphological and physiological disorders. Echocardiography is a method allowing to visualize structures of the heart using reflection of ultrasounds of 1.5–10 MHz frequency. Using the Doppler method allows to obtain important data concerning hemodynamic function of the heart. This method is commonly available and non-invasive. It does not require special preparation of the patient and there are no contraindications to perform it. However, the correct assessment of echocardiography results requires experience.

## Dimensions of the heart

Parameters assessed in echocardiography are end-diastolic and end-systolic diameters of the left ventricle (LVDD and LVSD, respectively) and the left atrial diameter (LAD). In most of the analyzed studies, there were no significant abnormalities in these dimensions of the heart observed in patients with psoriasis vulgaris [2, 12, 15, 16, 21] and psoriatic arthritis [39]. However, in a study conducted by Biyik et al. [6], there were symptoms of left ventricle’s hypertrophy observed in patients with psoriasis vulgaris. Hypertrophy occurred more frequently in patients suffering from psoriasis in comparison with control group (11.1 % in group of patients with psoriasis, 4.6 % in control group). Symptoms of the left ventricle’s hypertrophy were related to higher blood pressure in these patients. It is worth to notice that hypertension is a common disease occurring all over the world and it is an etiological factor of left ventricle’s hypertrophy. Gunes et al. [17] also found the echocardiographic signs of mild left ventricle’s hypertrophy in 3 out of 47 patients. Saricaoglu et al. [37] assessed heart’s dimensions in 21 patients with psoriatic arthritis using echocardiography. They observed slight difference in LVDD and LVSD in patients with psoriasis compared with control group (mean LVDD was 52.76 mm in patients with psoriasis, while it was 46.87 mm in control group). These differences were observed in older patients, patients with a longer course of psoriasis and psoriatic arthritis. In the study conducted by Gonzales-Juanatey et al. [15], left ventricle’s dimension in patients with psoriatic arthritis was within the normal values range, but mean left atrial diameter (LAD) was bigger compared with control group (35.9 and 34.3 mm, respectively). Eliakim-Raz et al. [11] observed more frequent occurrence of idiopathic cardiomyopathy in patients with psoriasis on the basis of their own observations and analysis of cases of patients hospitalized from 1980 to 2008. Based on the assessment of 2292 hospitalized patients suffering from psoriasis vulgaris and psoriatic arthritis, it was estimated that the frequency of idiopathic dilated cardiomyopathy in patients was 0.43 %. Potential hypotheses concerning relations between these diseases are the genetic influence and chronic inflammatory process with constant cytokines’ secretion [11].

## Ejection fraction

The assessment of systolic function of the left ventricle is one of the most important aims of performing echocardiography. It is based on the measurement of left ventricular ejection fraction (LVEF); range of the normal values is within 55–80 %. Systolic function impairment is a base for diagnosing congestive heart failure. In patients with psoriasis vulgaris and psoriatic arthritis, there were no systolic disorders of left ventricle observed by measuring the ejection fraction. LVEF was found to be normal in patients with psoriasis in numerous studies [2, 11, 12, 15, 16, 21, 39]. However, Saricaoglu et al. [37] observed LVEF below 55 % in 3 out of 21 patients with psoriatic arthritis. On the other hand, Zhao et al. [43] studies did not reveal any significant difference in systolic function as well as diastolic function of the left ventricle between patients with severe psoriasis and control group. However, using the speckle strain echocardiography in patients with psoriasis they demonstrated significantly changed myocardial deformation markers: transversal and longitudinal deformation. These markers were significantly correlated with an increased parathormone (PTH) concentration in blood. Observed markers alterations were not correlated with brain natriuretic peptide (BNP) concentration in blood, which was similar in patients with psoriasis and in control group. What is more, Bulbul Sen et al. showed significantly higher prevalence of left ventricle systolic dyssynchrony (assessed in tissue Doppler echocardiography) in psoriatic patients with normal ejection fractions and normal QRS width in ECG in comparison with the control group (34 vs. 6 %, respectively) [8]. Loss of ventricular contraction coordination was previously described in patients with systolic heart failure, diabetes mellitus and myocardial infarction. Osto et al. [28] showed a significant reduction of coronary reserve in group of 56 young patients (mean age of 37 years) with severe psoriasis and without any standard cardiovascular risk factors by assessing the coronary blood flow at rest and after adenosine infusion in transthoracal echocardiography. According to the authors, this may indicate on alterations in the microcirculation in the coronary system, which is caused by the systemic inflammatory process related to psoriasis only. What is more, it is the surface of erythema, not the intensity of desquamation or infiltration, which was correlated with extent of the coronary reserve reduction.

## Parameters of diastolic function

Echocardiography is also a basic method of diastolic function of the heart assessment. In some patients, there is an isolated diastolic heart insufficiency observed. Diastolic dysfunction may be the first sign of heart diseases as well as metabolic diseases. Diastolic dysfunction is more frequently observed in elderly people. Assessment of diastolic function in echocardiography is based on measuring the mitral inflow parameters, which are isovolumic relaxation time (IVRT), early to late ventricular filling velocities ratio (E/A), early flow deceleration time (DT). There were no significant diastolic function disorders in patients with psoriasis vulgaris and psoriatic arthritis noticed on the basis of the analyzed studies [2, 12, 15, 21]. However, many authors notice the prevalence of occurrence of the subclinical left ventricle diastolic dysfunction among patients with psoriasis in comparison with healthy population. There was a significantly higher frequency of the left ventricle diastolic function disorders occurrence in comparison with control group (27.8 and 13.9 %, respectively) [6]. In the same study, there was a relation between diastolic dysfunction occurrence and psoriasis duration observed—the longer psoriasis history, the more frequent occurrence of left ventricle diastolic disorders. On the other hand, in study conducted by Simsek et al. [40], mean values of the analyzed parameters (DT and IVRT) were higher in group of patients with psoriasis in comparison with control group, but the frequency of these disorders was comparable in both groups. Similar subclinical features of diastolic dysfunction were observed in patients with psoriatic arthritis using Doppler echocardiography. In study conducted by Shang et al. [39], there were 94 patients with psoriatic arthritis examined. Among these patients, the researchers distinguished a group of patients without standard cardiovascular risk factors and a group of patients with these risk factors. In patients with standard risk factors, occurrence of subclinical left ventricle diastolic function disorders was found to be significantly higher (75 % of the group). Subclinical features of left ventricle dysfunction were found in 47.2 % of group of patients with no risk factors in medical history. Patients presenting echocardiographic features of left ventricle dysfunction were older at the time of the diagnosis of psoriatic arthritis, medical history of this disease was longer and frequency of occurrence of hypertension and lipid metabolism disorders was higher. However, there was no correlation of activity and severity of disease and diastolic function disorders noticed. It seems to be important to notice the presence of subclinical diastolic function disorders in patients with psoriatic arthritis with no other risk factors. Similar results on left ventricle diastolic dysfunction in patients with psoriatic arthritis were obtained in a study conducted by Rowe et al. [36]. In this research, there was IVRT prolongation observed in 63 % of patients. This abnormality was correlated neither with age nor with psoriasis duration. However, in another study [15] diastolic function disorders were found in 28 % of patients with psoriasis, but these disorders occurred with similar frequency in control group (24 %). Diastolic disorders were observed in 26.7 % of cases of psoriatic arthritis [33]. In this research, diastolic function disorders were correlated with psoriasis duration and presence of psoriatic arthritis. Gullu et al. [16] noticed slight abnormalities of E/A ratio, DT and IVRT among patients with psoriasis in comparison with control group. Saricaoglu et al. [37] also observed diastolic function impairment demonstrated by lowered E/A ratio (<1) in 11 out of 21 patients with psoriatic arthritis.

## Pulmonary artery blood pressure

Echocardiography is also used to measure blood pressure in the pulmonary artery. Value of this parameter is obtained by measuring maximal speed of regurgitation flow through pulmonary valve or tricuspid valve. In a few studies on this subject, the authors observe higher pulmonary artery blood pressure in patients with psoriasis vulgaris and psoriatic arthritis. Gunes et al. [17] found the presence of echocardiographic features of mild pulmonary hypertension in patients with psoriasis (SPAP 30-40 mmHg). Value of systolic blood pressure in the pulmonary artery was correlated with BMI, but there was no correlation between value of pulmonary artery blood pressure and age, sex, psoriasis duration and PASI. However, other authors did not find any evidence of link between psoriasis and pulmonary hypertension. In a study conducted by Gonzales-Juanatey et al. [15], mean value of diastolic pulmonary blood pressure in patients with psoriatic arthritis was 23.4 mmHg and it was lower when compared with control group (25.7 mmHg). In this study, there was no evidence of pulmonary hypertension among patients with psoriasis found, but in 8 % of the patients estimated diastolic pulmonary artery blood pressure was within the highest normal values (30–35 mmHg). Pulmonary hypertension in patients with psoriasis may be caused by the coexistence of chronic obstructive pulmonary disease (COPD). On the basis of the analysis of over 12 thousand patients with psoriasis and 24-thousand-person control group (Clalit Health Services), Dreiher et al. [10] stated that COPD is significantly more frequent among patients with psoriasis (OR 1.63, CI 1.47–1.81). What is more, in the logistic regression model link between psoriasis and COPD turned out to be significant after taking into consideration other factors: age, sex, socioeconomic status, smoking and obesity. Similar data referring to the American population were obtained by Pearce et al. [29]. The reasons for correlation between these diseases (psoriasis and COPD) are of various origins. Both diseases are the inflammatory states and in their course there is an increase of pro-inflammatory cytokines and B helper cells activity. The autoimmunological reaction towards organs containing elastin, such as skin and vessels, was observed in COPD [13]. COPD may be then an autoimmunological disease induced by smoking [1]. Both diseases may share the same etiopathogenetic factors—smoking and more frequent in both diseases metabolic syndrome, which contributes to maintaining the inflammatory state by inducing antibodies against oxidized lipoproteins [24].

## Valvular defects

Valvular defects in patients with psoriasis were analyzed in the oldest described works. In 1986, Pines et al. [31] studied the echocardiography results in patients with psoriatic arthritis. They found mitral valve prolapse to be more frequent in patients with psoriasis. Biyik et al. [6] estimated frequency of valvular defects in patients with psoriasis to be 40.7 %, while in control group it was 18.1 %. In another study, 26 % of patients with psoriasis presented valvular defects which were mitral, tricuspid and aortal insufficiency, whereas these defects were observed in 20 % of control group [15]. Among patients with psoriatic arthritis, aortal valve insufficiency was observed in 36.7 % of cases and mitral insufficiency in 16.7 % of cases [33].

## Aortic stiffness index

Aortic stiffness index (ASI) is an echocardiographic parameter used to assess the elastic features of the aorta. An abnormal, increased ASI is observed in patients with hypertension, ischemic heart disease, heart insufficiency, type 2 diabetes, chronic renal insufficiency. ASI is thought to be one of the predictive factors of cardiovascular morbidity and mortality. That is why this parameter is often used during echocardiography of patients with psoriasis. Ardic et al. [2] found ASI values to be higher in 58 patients with psoriasis (2.7) in comparison with control group (2.0). What is intriguing, there was a correlation between psoriasis duration and the ASI values. There was no correlation found between ASI value and psoriasis severity. What is more, there was a noticeably higher concentration of the inflammatory state marker (C-reactive protein—hs-CRP) in serum of patients with psoriasis. There were no other significant echocardiographic abnormalities found in this study. Similar results were obtained by Shang et al. in 2012 [38]. The study was conducted on 73 patients with psoriatic arthritis. Despite the lack of hypertension or left ventricle hypertrophy features, ASI value of these patients was higher in comparison with control group. The main factor related to the described abnormalities was psoriasis duration. These results are consistent with the study conducted by Bicer et al. [5], who also observed ASI value to be higher in 27 patients with psoriasis in comparison with control group (4.98 vs. 2.73). In this study, ASI value was correlated with PASI and psoriasis duration. However, Balta et al. did not find any correlation between aortic arterial stiffness and psoriasis duration and severity [4]. Results obtained so far are not consistent for many reasons. The authors present patients with various PASI (data in Table 1), some of the authors do not describe PASI value, PASI range in Shang et al. [38] study is 1.0–8.0, in Gunes [17] studies 0.4–34, in Simsek [40] and Biyik [6] studies 1.8–60. Some of the researchers conduct studies on various groups of patients with specific forms of psoriasis, like pustular psoriasis or palmoplantar psoriasis and erythroderma affecting whole skin surface [6, 37, 38, 40]; groups are then not homogenous. Not all the works contain the treatment methods descriptions, some groups are treated with methotrexate [2, 5, 15] and immunosuppressive agents, what makes choosing and comparing groups of patients a difficult task. However, correlation of echocardiography results and psoriasis demonstrated in the studies demands paying attention to cardiovascular diseases in patients with psoriasis and treating them adequately.

## Summary

To sum up, despite a number of studies on echocardiography results assessment in patients with psoriasis and psoriatic arthritis, results remain incoherent and sometimes contradictory. Irrefutably, there is a higher prevalence of left ventricle diastolic dysfunction in patients with psoriasis. Subclinical features of left ventricle diastolic function disorders are observed in 8–27 % of patients with psoriasis vulgaris and in 28–63 % patients with psoriatic arthritis. It seems worth noticing that diastolic function disorders are also observed in patients with no standard cardiovascular risk factors. In spite of generally well-preserved left ventricle systolic function, the episodes of systolic disorders were demonstrated using Speckle Tracking Echocardiography. What is more, the aorta elasticity disorders, more frequent occurrence of pulmonary hypertension, mitral and aortal valves insufficiency and left ventricle hypertrophy features with coexistence of hypertension were found in patients with psoriasis. It seems vital to conduct further studies to precisely assess the cardiovascular system function in patients with psoriasis as an increased cardiovascular morbidity and mortality is observed in this group.

Although there are no clearly defined indications concerning necessity of performing echocardiographic examination in patients with psoriasis, dermatologists should pay special attention to the possibility of cardiovascular disorders among their patients.
